# Domestic Environment of Multisensory Stimulation based on Snoezelen principles pilot study

**DOI:** 10.1002/alz70858_101698

**Published:** 2025-12-25

**Authors:** Beatriz Zima Borsari Mauricio, Rosezélia da Silva Batista, Marcia Maria Pires Camargo Novelli

**Affiliations:** ^1^ Child Behavior Institute of Miami., Miami, FL, USA; ^2^ Federal University of São Paulo (UNIFESP), Santos, São Paulo, Brazil

## Abstract

**Background:**

Multisensory Stimulation Interventions (MSI) aims to stimulate the senses such as sight, hearing, touch, taste, smell, vestibular and proprioceptive systems, which can be areas affected by dementia. Studies on MSI in the home environment, both for older adults with dementia and for their family caregivers, are scarce.

**Method:**

MSI in home environments was carried out in 8 sessions, lasting 1.5 hours and occurring weekly. The sample was allocated into two groups: Experimental and Control. The benefits of the intervention were assessed by objective outcome measures such as the Neuropsychiatric Inventory (NPI) and the Anxiety, Depression and Stress Scale (DASS‐21) through descriptive and comparative analyses considering pre‐ and post‐intervention and effect size.

**Result:**

The sample consisted of 10 families (older adults and their family caregivers). Comparing the pre‐ and post‐intervention through the NPI, there was an improvement of great magnitude in the EG in all the variables: Total score in the NPI (d Cohen = ‐1.07), Frequency (d Cohen = ‐1.33), Intensity (d Cohen = ‐1.45), Caregiver distress (d Cohen = ‐1.06) and quantity of behaviors (d Cohen = ‐1.14). In the DASS‐21 there was an improvement in the following variables: DASS‐21 total score (Cohen's *d* = ‐0.65), Anxiety (Cohen's *d* = ‐1.05) and Stress (Cohen's *d* = ‐0.30), a value of *p* ≤0.05 was adopted.

**Conclusion:**

The MSI intervention based on the principles of Snoezelen in home environments has a positive effect on reducing the frequency and intensity of BPSD in the older adults, as well as on the caregiver distress, anxiety and stress of the caregivers regarding these behaviors.

